# Buckling up in Singapore: residency and other risk factors for seatbelt non-compliance – a cross-sectional study based on trauma registry data

**DOI:** 10.1186/s12889-016-3080-3

**Published:** 2016-05-14

**Authors:** Ting Hway Wong, Gek Hsiang Lim, Khuan Yew Chow, Trauma Coordinators and Trauma Service Representatives, Nyi Nyi Zaw, Hai Van Nguyen, Hoong Chor Chin, Marcus Eng Hock Ong

**Affiliations:** Department of General Surgery, Singapore General Hospital, Outram Road, Singapore, 169608 Singapore; Health Promotion Board, 3 Second Hospital Ave, Singapore, 168937 Singapore; Department of Emergency Medicine, Singapore General Hospital, Outram Road, Singapore, 169608 Singapore; Duke-National University of Singapore Graduate Medical School, 8 College Rd, Singapore, 169857 Singapore; National University of Singapore, 10 Kent Ridge Crescent, Singapore, 119260 Singapore

**Keywords:** Seatbelt compliance, Immigrant behaviour, Singapore, Asia, Trauma registry

## Abstract

**Background:**

Seatbelt non-compliance is a problem in middle income countries, and little is known about seatbelt compliance in populations with a high proportion of non-residents. This study analyses the profile of seatbelt non-compliance in Singapore based on trauma registry data from five of the six public hospitals.

**Methods:**

This is a cross-sectional study of seatbelt compliance of patients aged over 18 years, attending the emergency departments of five public hospitals in Singapore after road collisions from 2011–2014. Seatbelt data was obtained from paramedic and patient history.

**Results:**

There were 4,576 patients studied. Most were Singapore citizens (83.4 %) or permanent residents (2.4 %), with the largest non-resident groups from Malaysia, India, and China. Overall seatbelt compliance was 82.1 %.

On univariate analysis, seatbelt compliance was higher in older patients (OR 1.02, 95 % CI 1.001–1.021, *p* < 0.0001); drivers, followed by front passengers (OR 0.65, 95 % CI 0.51–0.83, *p* < 0.0001), were more compliant than rear passengers (OR 0.08, 0.06–0.09, *p* < 0.0001); occupants of larger vehicle types (buses, heavy transport vehicles, minibuses and vans) were more non-compliant compared to occupants of private cars and taxis. Morning peak travel (0700 h-0900 h) and being a non-resident were other risk factors for non-compliance.

On multivariable analysis, older age (OR 1.01, 95 % CI 1.001–1.014, *p* = 0.03) was associated with compliance, while non-residents from China (OR 0.43, 95 % CI 0.18–0.99, *p* = 0.05), seat position (front passenger compared to driver, OR 0.64, 95 % CI 0.48–0.85, *p* = 0.002; rear passenger compared to driver, OR 0.067, 95 % CI 0.05–0.09, *p* < 0.0001), vehicle type (bus compared to car, OR 0.04, 95 % CI 0.017–0.11, *p* < 0.0001, van compared to car, OR 0.55, 95 % CI 0.36–0.83, *p* = 0.004), and travel at morning peak periods were independent predictors of seatbelt non-compliance.

When the sub-group of drivers was analysed, only vehicle type was a significant predictor of seatbelt compliance, with bus drivers least likely to be compliant to seatbelts (multivariable analysis, OR 0.057 compared to cars, 95 % CI 0.019–0.18, *p* < 0.0001).

**Conclusions:**

While overall seatbelt compliance in our study is high, efforts can be made to increase compliance for morning rush hour passengers, rear seat passengers, and occupants of buses, heavy transport vehicles, and vans or pickups.

## Background

Seatbelt non-compliance is a problem in middle income countries, with seatbelt use lagging behind vehicle ownership trends [1, 2]. Seatbelt compliance rates can be estimated by observational studies, self-report questionnaires, or from on-scene information for motor vehicle crash victims [3–7]. Understanding the profile of vehicle users who are non-compliant with seatbelt regulations has important implications for targeted safety campaigns and educational measures.

Singapore is a city-state with a land area of 719.1 km^2^ and a population of 5.5 million. The population is multiracial with the major ethnic groups being Chinese, Malay, and Indian, and with non-residents comprising almost 30 % of the population [8]. Seatbelt legislation for drivers and front passengers was passed in Singapore in 1973, and for rear passengers in 1993. As such, seatbelt compliance and installation of seat belts has been required in both the front and rear of cars and taxis as of 1993. Rear belts in private buses, mini-buses, and vans are mandatory in vehicles registered on or after April 2009 [9], and it is mandatory to use them where available. While there have been studies of seatbelt compliance in the region showing relatively low compliance with seatbelts [10, 11], especially for rear seat passengers [12], there have been no studies of seatbelt compliance in Singapore. Studies from the region have been observational or survey-based [10–20]. Pure observational studies without direct interview with the occupants cannot differentiate non-residents from residents, while self-report questionnaires may be difficult to administer to certain social and linguistic groups, including the transient migrant worker and non-resident population.

Motor vehicle crashes are the second highest cause of injury in Singapore after falls [21]. Among the motor vehicle crash victims, injuries to motor vehicle occupants are the second most common, after motorcyclist injuries. The goal of our study was to determine the prevalence and analyse the associated risk factors of patients who are non-compliant with seatbelts, with a view to target high-risk groups in future. Of particular interest in our setting of a high-non-resident population was whether residency status and country of origin affected seatbelt compliance.

## Methods

### Data source and data collection

Seatbelt compliance is one of the optional data collection fields collected in five of the six public hospitals participating in the Singapore National Trauma Registry (NTR), which was started in 2011 [21]. Data collection for the NTR is performed by teams of trained trauma data coordinators based at each public hospital, with data cleaning, data completeness, data quality, and inter-rater audits performed annually; inclusion criteria and data collection details can be found in the annual report and previous publications [21, 22]. There is a single national emergency ambulance number and all hospitals that receive Singapore Civil Defence Force ambulances (i.e. all public hospitals) provide data for the NTR [23].

De-identified data from five of the six adult public hospitals contributing data to the NTR were pooled. Three hospitals collected seatbelt compliance data from 2011, one started in 2012, and one started in 2013. The remaining public hospital, which has the lowest patient volume of the six hospitals, did not collect data for the optional field on seatbelt compliance. Two of the hospitals routinely collect seatbelt compliance data for all patients who present to the hospital, while three collected data only for moderately and severely injured patients (injury severity score of 9 and above). Seatbelt data was primarily entered based on paramedic and patient history, with a minority of cases being inferred from the mechanism of injury (e.g. patient found unbelted on the floor of the vehicle without vehicle rollover). Demographic data, use of seatbelt, position in vehicle (driver, front passenger, or rear passenger), vehicle type (private car, taxi, bus, mini-bus, van/pick-up truck, or heavy goods vehicle), alcohol (documented clinically and/or confirmed on toxicology), pregnancy status where documented, date and time of injury presentation, and whether the injury was work-related were extracted from the registry. The de-identified datasets from the hospitals were merged with the National Trauma Registry data to obtain the demographic information of the subjects. The merged dataset was analysed by a statistician in the National Registry of Diseases Office (NRDO) that manages NTR, aggregated numbers were then released to the research/study team for further interpretation.

### Study population

Vehicle occupants aged 18 and above attending the emergency departments of participating institutions after a motor vehicle collision were included in the study. Patients without known seatbelt information were excluded from analysis.

As the availability of rear seatbelts in heavy transport vehicles, vans, trucks, mini-buses, and buses depends on the date of vehicle registration (only mandatory for vehicles registered after the law was passed in 2009) [9], patients who were rear passengers in these vehicles were excluded from our analysis, as it was not possible to distinguish whether the lack of compliance in these patients was due to the lack of availability of seatbelts or patients’ behaviour.

### Study design

This was a cross-sectional study based on prospectively collected data from January 2011 to December 2014, of patients aged over 18 years, attending the emergency departments of five public hospitals in Singapore after road collisions. Seatbelt data was obtained from paramedic and patient history during the admission for the incident.

The associations between seatbelt compliance and age, gender, race, residency status, position in vehicle, vehicle type, alcohol, pregnancy, time of day, weekday versus weekend, and whether injury was work-related were examined. In view of the different inclusion criteria for the various contributing hospitals, the hospital was included as a covariate in the multivariable model.

### Outcome measure

The outcome of interest was seatbelt compliance.

### Statistical analysis

Patient characteristics at baseline were summarised by mean (standard deviation), median (inter-quartile range), or frequency (%) as appropriate. Chi-square tests and Fisher’s exact test were performed to evaluate associations between the outcomes of interest and other categorical predictors of interest, while t-test was used for age, after testing for normality and homogeneity of variance. Univariate logistic regression was used to analyse association with seatbelt compliance. Multivariable analysis was performed for factors that were significant at the 10 % level in the univariate analysis. Factors that were not significant on univariate analysis, but known to be risk factors in the literature, were included in the multivariable model. Sensitivity analysis excluding these variables was performed. Patients with missing seatbelt information were excluded from the main model. Stata 13.0 was used.

## Results

### Descriptive analysis

From 2011 to 2014, there were 4,911 patients with documented seatbelt status in the five institutions. There were 3996 vehicle occupants whose seatbelt status was not available. Of the patients with documented seatbelt status, the following patients were excluded: 96 patients coded as rear seat passengers in a bus, 19 patients coded as rear seat passengers in a mini-bus (defined as fewer than 20 passengers), 86 patients coded as rear seat passengers in a van or pickup, and 134 patients coded as rear passengers in heavy goods vehicles. Seatbelt compliance was only 6.9 % in these excluded patients.

Of the remaining 4,576 patients studied, the median age was 40.7 years, with males accounting for nearly two-thirds of the sample (64.4 %). The majority were Singapore citizens (83.4 %) or permanent residents (2.4 %), with the largest non-resident groups coming from Malaysia, India, and China. Overall seatbelt compliance in the study population was 82.1 %. Summary statistics are presented in Table 1.

**Table 1:** Summary Statistics

		Number (%)/Mean (SD)
	Seatbelt Compliance	3759 (82.1)
Demographics		
	Age	40.7 (SD 14.1)
	Males	2949 (64.4)
Country of Residence	Singapore	3928 (85.8)
	Malaysia	122 (2.7)
	India	51 (1.1)
	China	45 (1.0)
	Other Nationalities	430 (9.4)
Vehicle Type		
	Bus (capacity of 20 or more passengers)	23 (0.5)
	Heavy Transport Vehicle	282 (6.2)
	Minibus (less than 20 passengers)	23 (0.5)
	Private Car	2706 (59.1)
	Taxi	890 (19.4)
	Van or Pickup	248 (5.4)
	Unknown	404 (8.8)
Seat Position		
	Driver	2853 (62.3)
	Front Passenger	836 (18.3)
	Rear Passenger	798 (17.4)
	Unknown	89 (1.9)
Other Associated Risk Factors		
	Alcohol Use	73 (1.6)
	Pregnancy	3
	Work-related	81 (1.8)
Time of Arrival	0700–0959 h	406 (8.9)
	1000–1659 h	1647 (36.0)
	1700–1959 h	744 (16.3)
	2000–0659 h	1779 (38.9)
Day of Arrival	Weekdays	3261 (71.3)

### Univariate analysis

Seatbelt compliance was higher in older patients; drivers, followed by front passengers, were more compliant than rear passengers, while occupants of larger vehicle types were more non-compliant compared to occupants of private cars (Table 2).

**Table 2:** Factors affecting Seatbelt Compliance

		Seatbelt Compliance	Non-compliant [Number (%)/Mean (SD)]	Compliant [Number (%)/Mean (SD)]	Odds Ratio	95 % CI	*P*
Demographics		Age	38.3 (SD 15.0)	41.2 (SD13.8)	1.02	1.001–1.021	<0.0001
		Males	406 (13.67)	2563 (86.33)	2.17	1.86–2.53	<0.0001
		Females	411 (25.58)	1196 (74.42)	1 (Ref)		
Country of Residence		Singapore	649 (16.52)	3279 (83.48)	1 (Ref)		<0.0001
		Malaysia	14 (11.48)	108 (88.52)	1.53	0.87–2.68	
		India	7 (13.73)	44 (86.27)	1.24	0.56–2.77	
		China	13 (28.89)	32 (71.11)	0.49	0.25–0.93	
		Other Nationalities	134 (31.16)	296 (68.84)	0.44	0.35–0.55	
Vehicle Type		Bus (capacity of 20 or more passengers)	15 (65.22)	8 (34.78)	0.09	0.04–0.22	<0.0001
		Heavy Transport Vehicle	30 (10.64)	252 (89.36)	1.43	0.96–2.12	
		Minibus (less than 20 passengers)	4 (17.39)	19 (82.61)	0.81	0.27–2.38	
		Private Car	393 (14.52)	2313 (85.48)	1 (Ref)		
		Taxi	231 (25.96)	659 (74.04)	0.48	0.40–0.58	
		Van or Pickup	33 (13.31)	215 (86.69)	1.11	0.76–1.62	
		Unknown vehicle	111 (27.48)	293 (72.52)	0.45	0.35–0.57	
Seat Position		Driver	245 (8.59)	2608 (91.41)	1 (Ref)		<0.0001
		Front Passenger	105 (12.56)	731 (87.44)	0.65	0.51–0.83	
		Rear Passenger	441 (55.26)	357 (44.74)	0.08	0.06–0.09	
		Unknown position	26 (29.21)	63 (70.79)	0.23	0.14–0.37	
Other Associated Risk Factors	Alcohol use	Alcohol Use (clinical or toxicological)	13 (17.81)	60 (82.19)	1.002	0.55–1.83	0.994
		No alcohol use	777 (17.84)	3578 (82.16)	1 (ref)		
	Pregnancy	Pregnant	2 (66.67)	1 (33.33)	0.17	0.02–1.85	0.202
		Not pregnant or status unknown, female aged below 50	209 (18.54)	918 (81.46)	1 (ref)		
	Work-related	Work-related	7 (8.64)	74 (91.36)	1 (ref)		0.029
		Not Work-related	810 (18.04)	3681 (81.96)	2.33	1.07–5.07	
Time of Arrival		0700–0959 h	92 (22.66)	314 (77.34)	1 (ref)		0.040
		1000–1659 h	300 (18.21)	1347 (81.79)	1.32	1.01–1.71	
		1700–1959 h	126 (16.94)	618 (83.06)	1.44	1.06–1.94	
		2000–0659 h	299 (16.81)	1480 (83.19)	1.45	1.11–1.89	
		Weekdays	566 (17.36)	2695 (82.64)	1 (ref)		0.167
		Weekends	251 (19.09)	1064 (80.91)	0.89	0.76–1.05	

Females were more likely to be non-compliant. Non-residents were also more likely to be non-compliant. Non-resident groups were further assessed by individual nationalities, and divided into nationalities comprising more than 1 % of the study population (citizens of Malaysia, India, and China), with the remaining nationalities grouped together (“others”). While work-related injuries were not significant on overall univariate analysis, a closer inspection of the subgroup of drivers who were non-compliant showed that they were mostly work-related large vehicle drivers.

While there was no difference between weekday and weekend compliance, morning rush hour crashes yielded more patients who were un-belted. The time-bands were chosen to reflect morning and evening office commute peak hour timings, daytime non-peak and night-time non-peak. Histograms of the compliance levels across the 24-h time-period showed that compliance levels were fairly consistent within each of the four time-bands.

Patients from hospitals where data on seatbelt compliance was also collected for patients with only minor injuries were more likely to have worn a seatbelt, which is consistent with the protective effect of seatbelts.

### Multivariable analysis

Younger age, seat position, vehicle type, and travel at morning peak periods were independent predictors of seatbelt non-compliance (Table 3). The only factor significant on univariate analysis, but not multivariable analysis, was gender. As the number of pregnant women in the study was low and not significant on univariate analysis, this factor was omitted from multivariable analysis.

**Table 3:** Multivariable Logistic Regression – Likelihood of Seatbelt Compliance

	Seatbelt Compliance	Odds Ratio	95 % CI	*P*
Demographics				
	Age	1.01	1.001–1.014	0.03
	Male (vs Female)	1.13	0.90–1.41	0.31
Country of Residence				
	Singapore	1 (Ref)		
	Malaysia	1.48	0.73–2.99	0.28
	India	0.85	0.30–2.37	0.75
	China	0.43	0.18–0.99	0.05
	Other Nationalities	0.93	0.69–1.25	0.62
Vehicle Type				
	Bus (capacity of 20 or more passengers)	0.04	0.017–0.11	<0.0001
	Heavy Transport Vehicle	0.62	0.39–0.97	0.04
	Minibus (less than 20 passengers)	0.39	0.13–1.18	0.09
	Private Car	1 (Ref)		
	Taxi	0.94	0.74–1.19	0.59
	Van or Pickup	0.55	0.36–0.83	0.004
Seat Position				
	Driver	1 (Ref)		
	Front Passenger	0.64	0.48–0.85	0.002
	Rear Passenger	0.067	0.05–0.09	<0.0001
Other Associated Risk Factors				
	Alcohol use (vs no alcohol use)	0.89	0.42–1.89	0.77
	Work-related (vs non-work-related)	1.22	0.48–3.06	0.68
Time of Arrival				
	0700–0959 h	1 (Ref)		
	1000–1659 h	1.26	0.89–1.77	0.19
	1700–1959 h	1.40	0.95–2.07	0.09
	2000–0659 h	1.46	1.03–2.07	0.03
	Weekdays	1 (Ref)		
	Weekends	0.96	0.78–1.18	0.67

Sensitivity analyses included: exclusion of all factors that were not significant on univariate analysis, re-categorisation of non-citizen permanent residents as non-residents, and inclusion of patients with missing vehicle type and seat position information in the multivariable models. These models yielded similar results.

### Sub-group analysis of drivers

We further conducted a sub-group analysis of drivers (Table 4). Overall compliance was high, with 91.4 % of the 2,853 drivers wearing seatbelts. On univariate analysis, only vehicle type (*p* < 0.0001) and non-resident status (2360/2580 residents compliant, compared to 130/152, *P* = 0.01) were significant. However, when non-residents were grouped by citizenship (Malaysia, China, India and others), the compliance rate for drivers for these three countries was slightly higher than Singapore residents (96.6 % for the 59 drivers from Malaysia, 100 % for the 21 drivers from China, 96.0 % for the 25 drivers from India, compared to 91.5 % for the Singapore resident drivers), with the “other nationalities” contributing to the low non-resident compliance rate.

**Table 4:** Seatbelt Compliance of Drivers

	Seatbelt Compliance	Non-compliant [Number (%)/Mean (SD)]	Compliant [Number (%)/Mean (SD)]	OR	95 % CI	*P*
Demographics	Age	40.5 (SD 13.9)	42.2 (SD 13.2)	1.01	0.99–1.02	0.06
	Males	209 (8.94)	2129 (91.06)	0.77	0.53–1.11	0.15
	Females	36 (6.99)	479 (93.01)	1 (Ref)		
Country of Residence	Singapore	220 (8.53)	2360 (91.47)	1 (Ref)		0.06
	Malaysia	2 (3.39)	57 (96.61)	2.24	0.30–16.62	
	India	1 (4)	24 (96)	2.66	0.64–10.96	
	China	0 (0)	21 (100)	NA		
	Other Nationalities	22 (13.1)	146 (86.9)	0.62	0.39–0.99	
Vehicle Type	Bus (capacity of 20 or more passengers)	9 (52.94)	8 (47.06)	0.07	0.03–0.19	<0.0001
	Heavy Transport Vehicle	20 (9.09)	200 (90.91)	0.82	0.50–1.35	
	Minibus (less than 20 passengers)	2 (9.52)	19 (90.48)	0.78	0.18–3.40	
	Private Car	132 (7.62)	1601 (92.38)	1 (Ref)		
	Taxi	33 (6.61)	466 (93.39)	1.16	0.78–1.72	
	Van or Pickup	21 (11.35)	164 (88.65)	0.64	0.39–1.05	

On multivariable analysis, compared to car drivers, drivers of buses were 17.4 times more likely to be non-compliant with seatbelts (*p* < 0.0001), drivers of heavy transport vehicles were 1.9 times more likely to be non-compliant with seatbelts (*p* = 0.03), and drivers of vans or pickups 1.7 times more likely to be non-compliant with seatbelts (*p* = 0.06). None of the other factors were significant (Table 5).

**Table 5:** Multivariable Logistic Regression – Likelihood of Seatbelt Compliance, Drivers Only

	Seatbelt Compliance	Odds Ratio	95 % CI	*P*
Demographics				
	Age	1.01	0.99–1.02	0.39
	Males	0.95	0.63–1.42	0.80
Country of Residence				
	Singapore	1 (Ref)		
	Malaysia	1.70	0.22–12.92	0.61
	India	0.77	0.09–6.51	0.81
	China	NA		
	Other Nationalities	1.05	0.60–1.85	0.86
Vehicle Type				
	Bus (capacity of 20 or more passengers)	0.057	0.02–0.18	<0.0001
	Heavy Transport Vehicle	0.53	0.30–0.95	0.03
	Minibus (less than 20 passengers)	0.83	0.19–3.68	0.81
	Private Car	1 (Ref)		
	Taxi	1.18	0.76–1.83	0.46
	Van or Pickup	0.60	0.35–1.01	0.06

### Effect of seatbelt compliance on injury severity

We compared the severity of injuries of those who were not compliant to the severity of those who were compliant, stratified by the key risk factors. The seatbelt-compliant group had less severe injuries. The results are presented in Table 6.

**Table 6:** Injury Severity by Seatbelt Compliance

	Injury Severity Score (ISS)	Compliant [Number (%)/Mean (SD)]	Non-Compliant [Number (%)/Mean (SD)]	*P*
Total Study Population	ISS <9	1205 (88.54)	156 (11.46)	<0.0001
	ISS 9–15	152 (80)	38 (20)	
	ISS > 15	2402 (79.4)	623 (20.6)	
Demographics				
Age				
	ISS <9	40.2 (SD 13.3)	36.1 (SD 14.3)	0.0003
	ISS 9–15	45.9 (SD 15.8)	51.9 (SD 17.3)	0.04
	ISS > 15	41.4 (SD 13.8)	38.0 (SD 14.6)	<0.0001
Male				<0.0001
	ISS < 9	773 (92.35)	64 (7.65)	
	ISS 9–15	115 (85.82)	19 (14.18)	
	ISS > 15	1675 (83.83)	323 (16.17)	
Country of Residence				
Singapore				<0.0001
	ISS < 9	992 (89.53)	116 (10.47)	
	ISS 9–15	130 (83.87)	25 (16.13)	
	ISS > 15	2157 (80.94)	508 (19.06)	
Malaysia				0.15
	ISS < 9	86 (90.53)	9 (9.47)	
	ISS 9–15	5 (100)	0 (0)	
	ISS > 15	17 (77.27)	5 (22.73)	
India				0.52
	ISS < 9	38 (88.37)	5 (11.63)	
	ISS 9–15	2 (66.67)	1 (33.33)	
	ISS > 15	4 (80)	1 (20)	
China				0.60
	ISS < 9	28 (73.68)	10 (26.32)	
	ISS 9–15	2 (50)	2 (50)	
	ISS > 15	2 (66.67)	1 (33.33)	
Other Nationalities				0.05
	ISS < 9	61 (79.22)	16 (20.78)	
	ISS 9–15	13 (56.52)	10 (43.48)	
	ISS > 15	222 (67.27)	108 (32.73)	
Vehicle Type				
Bus (capacity of 20 or more passengers)				0.20
	ISS < 9	4 (57.14)	3 (42.86)	
	ISS 9–15	0 (0)	3 (100)	
	ISS > 15	4 (30.77)	9 (69.23)	
Heavy Transport Vehicle				0.16
	ISS < 9	139 (92.67)	11 (7.33)	
	ISS 9–15	18 (85.71)	3 (14.29)	
	ISS > 15	95 (85.59)	16 (14.41)	
Minibus (less than 20 passengers)				0.06
	ISS < 9	4 (100)	0 (0)	
	ISS 9–15	0 (0)	1 (100)	
	ISS > 15	15 (83.33)	3 (16.67)	
Private Car				<0.0001
	ISS < 9	755 (91.18)	73 (8.82)	
	ISS 9–15	93 (84.55)	17 (15.45)	
	ISS > 15	1465 (82.86)	303 (17.14)	
Taxi				0.36
	ISS < 9	205 (77.07)	61 (22.93)	
	ISS 9–15	25 (69.44)	11 (30.56)	
	ISS > 15	429 (72.96)	159 (27.04)	
Van or Pickup				0.005
	ISS < 9	73 (96.05)	3 (3.95)	
	ISS 9–15	16 (94.12)	1 (5.88)	
	ISS > 15	126 (81.29)	29 (18.71)	
Seat Position				
Driver				<0.0001
	ISS < 9	820 (97.16)	24 (2.84)	
	ISS 9–15	98 (89.91)	11 (10.09)	
	ISS > 15	1690 (88.95)	210 (11.05)	
Front Passenger				0.041
	ISS < 9	261 (91.26)	25 (8.74)	
	ISS 9–15	41 (89.13)	5 (10.87)	
	ISS > 15	429 (85.12)	75 (14.88)	
Rear Passenger				0.005
	ISS < 9	124 (53.68)	107 (46.32)	
	ISS 9–15	13 (38.24)	21 (61.76)	
	ISS > 15	220 (41.28)	313 (58.72)	
Time of Arrival				
0700–0959 h				<0.0001
	ISS < 9	126 (92.65)	10 (7.35)	
	ISS 9–15	15 (75)	5 (25)	
	ISS > 15	173 (69.2)	77 (30.8)	
All other times				<0.0001
(1000–1659 h	ISS < 9	1079 (88.08)	146 (11.92)	
1700–1959 h	ISS 9–15	137 (80.59)	33 (19.41)	
2000–0659 h)	ISS > 15	2229 (80.32)	546 (19.68)	

## Discussion

Our study is the first study of risk factors for seatbelt compliance in Singapore using national injury data, and provides insights into the vulnerable groups that could be targeted in our injury prevention strategies. Similar to other studies in the international and regional literature [4–6, 10–13, 24], older occupants and drivers of cars or taxis were more compliant with seatbelts, compared to occupants of buses, minibuses, heavy transport vehicles, vans or pickups. Rear seat passengers were much less likely to be compliant, and this is likely an underestimate since we only included the rear seat passengers of cars and taxis. Morning was the period with the lowest seatbelt compliance, a similar finding to another study in Asia [14].

As driver behaviour is known to affect compliance of the rest of the vehicle occupants [14], and as some studies only looked at driver behaviour [5, 10, 13, 15, 25], our subgroup analysis of drivers yielded some additional insights. There were overall low compliance rates among drivers of buses, mini-buses, heavy goods vehicles, and vans or pickups, similar to a recent study in Thailand [10]. The numbers of some of these categories (buses, mini-buses) in our study were low. However, as these would be professional drivers, who spend a lot of time on the road, they should still be a target for seatbelt compliance. Similar efforts should be made for drivers of heavy goods vehicles and vans or pickups, as they comprise more than 10 % of our study population.

We did not find any effect of alcohol, pregnancy, or gender in our study. It is not standard practice in Singapore to screen for blood toxicology in our hospitals unless requested by the police. Consequently, patients with a positive alcohol status in our registry include those documented clinically to have consumed alcohol (in the history or the physical examination), as well as those with blood alcohol levels requested by the police. Hence, alcohol status includes patients with mild alcohol levels below the legal limit, who might still be compliant with seatbelts. Pregnant patients in some studies have been shown to be less likely to be compliant with seatbelts [26–28], but there were too few pregnant women in our study. Many studies find males more likely to be non-compliant, but we did not find that in our study.

Studies examining immigrant health behaviours vary in their findings, depending on which health behaviour is studied [29, 30]. One interesting finding in our study is how non-Singaporean residents behave in Singapore. The main non-Singaporean patient groups (Malaysia, India, and China) all displayed higher overall seatbelt compliance than reported in surveys or observational studies conducted in these three countries [11–20]. The number of Singapore permanent residents in our study population was too low to compare permanent residents and non-residents from the same country. While our study numbers are not high, this suggests that living in a high-compliance country does affect behaviour. An alternative explanation is that the immigrant populations in Singapore are different from the vehicle occupants in their countries, for example, more educated, or more safety-conscious.

Overall compliance rate in our dataset (76.8 %) was higher than similar studies using trauma registry data conducted in North America and Europe [4–6], and even higher in drivers (91.4 %). Our study suggests that non-resident drivers from countries other than the three major non-resident countries of origin (Malaysia, China, and India) could be targeted for compliance. Further studies specifically targeting non-resident drivers could be undertaken, for example, surveys in the languages of these other nationalities, and additional information such as reasons for non-compliance could be obtained in these surveys. With regards to the non-drivers, passengers who are non-residents, particularly the non-driver passengers from China, could also be targeted. While the additional healthcare and societal costs of seatbelt non-compliance is beyond the scope of this paper, the significantly higher injury severity scores in the non-compliant patients suggest that these injuries would have incurred a lower societal and healthcare cost if seatbelt compliance were higher in our study population.

One major limitation of our study is that trauma registry data is only collected for patients brought to hospital, and is hence biased towards injured patients who would be less compliant to seatbelts, compared to survey-based or observational studies [31]. This method also depends on fitness of the patient for interview or documentation in the paramedics’ notes. A reliable patient history would be biased towards less injured patients. Similarly, paramedic documentation in severely-injured patients would focus on the severity of injuries and timely transfer to hospital, rather than providing information on seatbelt compliance. Another limitation is that patients may feel compelled to over-report their compliance, for example, due to fear of getting less insurance compensation or from prosecution. Nevertheless, given the relatively large number of patients in our study, collected across multiple sites, this is an important source of information to identify potential target groups for seatbelt compliance and injury prevention [32].

## Conclusion

While overall seatbelt compliance in our study is high, efforts can be made to further increase compliance for morning rush hour passengers, rear seat passengers, and occupants of buses, heavy transport vehicles, and vans or pickups. Non-resident vehicle occupants from Malaysia, India, and China have higher seatbelt compliance in Singapore than in studies conducted in their home countries.

### Ethics approval and consent to participate

Ethical approval was given by the first author’s (Singapore General Hospital) Institutional Review Board and all data was de-identified and analysed on password-protected computers. Consent was not obtained because information was anonymized and de-identified prior to analysis, as per NRDO protocol.

### Availability of data and materials

The data was obtained from a third party, the National Trauma Registry, established by Singapore’s Ministry of Health. Data are available from the National Registry of Diseases Office in Singapore for researchers who meet the criteria for access to confidential data. Details are available at https://www.nrdo.gov.sg/data-request/faq.

## Abbreviations

NRDO: National Registry of Diseases Office
NTR: Singapore National Trauma Registry
